# Microphytobenthos primary production estimated by hyperspectral reflectance

**DOI:** 10.1371/journal.pone.0197093

**Published:** 2018-05-14

**Authors:** Vona Méléder, Bruno Jesus, Alexandre Barnett, Laurent Barillé, Johann Lavaud

**Affiliations:** 1 Mer Molécules Santé (MMS)–EA 21 60, Université de Nantes, Nates, France; 2 BioISI–Biosystems & Integrative Sciences Institute, Campo Grande University of Lisboa, Faculty of Sciences, Lisboa, Portugal; 3 Littoral Environnement et Sociétés (LIENSs)–UMR 7266, CNRS/Université de La Rochelle, Institut du Littoral et de l’Environnement, 2 rue Olympe de Gouges, La Rochelle, France; 4 Botany and Plant Science–National University of Ireland, Galway, Ireland; 5 Takuvik–UMI 3376, CNRS/Université Laval, Département de Biologie, Pavillon Alexandre Vachon, Québec, Canada; CNRS, FRANCE

## Abstract

The use of remote sensing techniques allows monitoring of photosynthesis at the ecosystem level and improves our knowledge of plant primary productivity. The main objective of the current study was to develop a remote sensing based method to measure microphytobenthos (MPB) primary production from intertidal mudflats. This was achieved by coupling hyperspectral radiometry (reflectance, *ρ* and second derivative, *δδ*) and PAM-fluorometry (non-sequential light curves, NSLC) measurements. The latter allowed the estimation of primary production using a light use efficiency parameter (LUE) and electron transport rates (ETR) whereas ρ allowed to estimate pigment composition and optical absorption cross-section (a*). Five MPB species representative of the main growth forms: epipelic (benthic motile), epipsammic (benthic motile and non motile) and tychoplanktonic (temporarily resuspended in the water column) were submitted to increasing light intensities from dark to 1950 μmol photons.m^-2^.s^-1^. Different fluorescence patterns were observed for the three growth-forms and were linked to their xanthophyll cycle (de-epoxydation state). After spectral reflectance measurements, a* was retrieved using a radiative transfer model and several radiometric indices were tested for their capacity to predict LUE and ETR measured by PAM-fluorometry. Only one radiometric index was not species or growth-form specific, i.e. *δδ*_496/508_. This index was named MPB_LUE_ and could be used to predict LUE and ETR. The applicability of this index was tested with simulated bands of a wide variety of hyperspectral sensors at spectral resolutions between 3 and 15 nm of Full Width at Half Maximum (FWHM).

## Introduction

Microphytobenthos (MPB) assemblages are composed of photosynthetic bacteria and microalgae that colonize benthic sediments. Typically, diatoms are the dominant microalgae group forming golden-brown biofilms at the sediment surface during low tides [1–3]. These biofilms exhibit very high primary productivity rates that can result in contributions of up to 50% of the total estuarine autochtonous primary production [4] and provide essential ecosystem services, e.g. food sources for various trophic webs, sediment stabilization via exopolysaccharide secretion (EPS) cohesion, mediation of nutrients fluxes [2,5].

MPB spatial and temporal variability is a constraint for large-scale assessments of MPB biomass and primary production making the estimation of its contribution at the ecosystem level often limited to discrete stations, and then extrapolated to the whole mudflat. Most techniques used to assess MPB primary production rates require single point *in situ* measurements which are often inadequate to capture the spatial variability at the ecosystem level (for a review see [6]). Regarding MPB biomass, synoptic information have been obtained by time-consuming extensive field sampling campaigns [7], or using remote sensing technology [8].

Currently, remote sensing studies have mainly focused on quantifying MPB biomass [9–14] and no algorithm exists yet for estimating MPB primary production from remote sensing imagery. However, monitoring photosynthesis from Space, has been recently proposed for land resources (for a review see [15]. It is a great challenge to improve our knowledge of the main drivers and resources constraints of plant or algal productivity and it is needed for predicting impacts of climate change [10,15] and for the management of costal ecosystems [9,13]. The main objective of the current study is to develop a method to estimate MPB primary production directly from hyperspectral imagery in the visible domain. It is based on pigment absorption changes that are detected in reflectance spectra due to photosynthetic capacity changes as a response to changes in light environment.

This was achieved by coupling PAM-fluorometry measurements for estimating primary production [16–18], and spectroradiometry to measure reflectance spectra of five MPB species representative of the main growth forms. Growth-forms are known to strongly affect eco-physiological responses to light exposure: non photochemical quenching (NPQ) and xanthophyll cycle (XC) patterns [19–23]. Epipelic (moving freely between sediment particles) and epipsammic species (living in close association with individual sand grains) show respectively low and high NPQ values and XC efficiency during high light exposure, whereas tychoplanktonic species show a light response similar to epipelon [19]. Because of these different eco-physiological response features, different radiometric indices are expected to estimate primary production for the different growth forms (i.e. epipelon/tychoplankton *vs*. epipsammon). We propose here the first spectral index based on MPB growth forms spectral properties for estimating primary production via electron transport rate (ETR). Simulating airborne and satellite hyperspectral sensors in the VIS-NIR domain using generic bandwidth, this index offers promising prospects for global primary productivity assessment of intertidal MPB biofilms.

## Materials and methods

### Diatoms culturing

**Fig 1:**
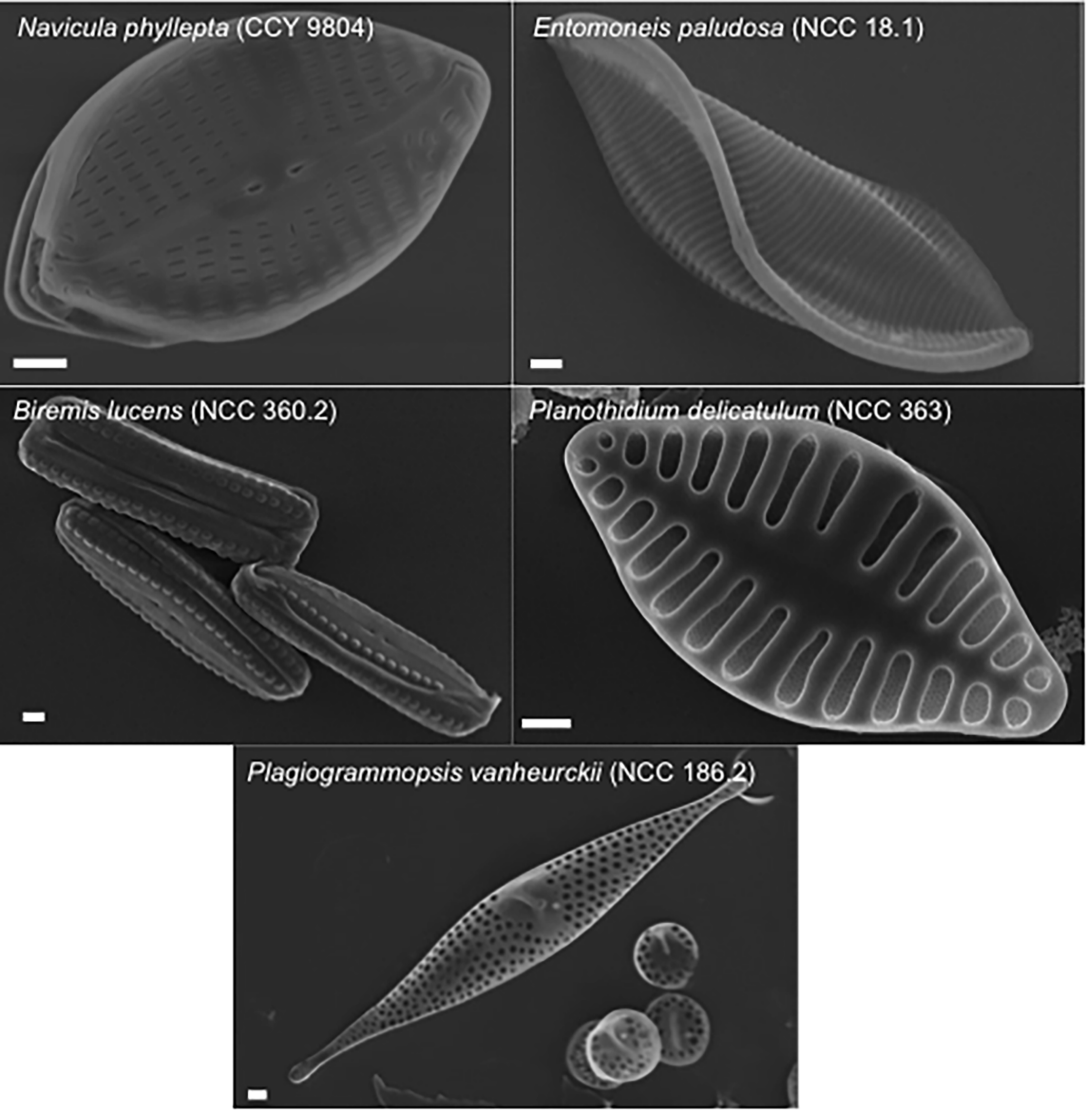
Specimens of the five diatom species viewed with scanning electron microscopy. Scale bars represent 1 μm. Credits Nantes Culture Collection (NCC). *Navicula phyllepta* and *Entomoneis paludosa* are epipelic; *Biremis lucens* and *Planothidium delicatulum* are epipsammic; *Plagiogrammopsis vanheurckii* is tychoplanktonic.

Five diatoms species were selected from a previous study [19] to carry out spectroradiometric and PAM-fluorescence measurements. These species were isolated from natural MPB biofilms and are kept in the Nantes Culture Collection (NCC WDCM 856) (Fig 1). *Navicula phyllepta* (CCY 9804) and *Entomoneis paludosa* (NCC 18.1) are epipelic motile species; *Biremis lucens* (NCC 360.2) and *Planothidium delicatulum* (NCC 363) are epipsammic, respectively motile and non-motile species; and *Plagiogrammopsis vanheurckii* (NCC 186.2) is a tychoplanktonic species. All diatoms were grown in batch cultures at 20°C in sterile artificial F/2 medium [24], at 20 μmol photon.m^-2^.s^-1^ and a 16h:8h light:dark photoperiod. Cultures were acclimated during 2 weeks before experiments. Diatom suspensions were concentrated to a final concentration of 10 mg Chl*a*.L^-1^ before each experiment. For further details, see [19].

### Light use efficiency (LUE) and relative electron transport rate (rETR) estimation by PAM-fluorometry

PAM fluorescence measurements were performed with a Diving-PAM fluorometer (Walz, Effeltrich, Germany) on a 2.5 ml cuvette stirred and 20°C controlled diatom suspension [25]. Suspension stirred was the selected configuration for these experimentations, rather than cells deposited on a surface, imitating natural biofilm, to enhance the illumination of cells during measurement. This choice avoided confusing physiological responses due to self-shading or epipelic migration movements. These controlled conditions allowed to measure for each species: minimum fluorescence yield (*F*_*o*_), maximum fluorescence yield (*Fm*) and maximum photosystem II (PSII) quantum yield (*Fv/Fm*) after 15 min dark-adaptation with a saturating pulse of 3600 μmol photons.m^-2^.s^-1^ (duration 400 ms). This was followed by non-sequential light curve (NSLC) measurements [26] using continuous light (KL-2500 lamp, Schott, Mainz, Germany) applied for 5 min at 9 light intensities (E) (48–1950 μmol photons.m^-2^.s^-1^); a new diatom suspension was used for each light intensity. NSLCs methodology was selected for these experimentations rather than RLCs (rapid light curve) for which the same suspension is used for at all light levels. Thus, NSLCs avoid the effects of light history and light dose accumulation in photosynthetic responses. At the end of each light level, the minimum fluorescence yield in light adapted state (*F’*) was measured and, with a saturating pulse, the maximum fluorescence yield in light acclimated state (*F*_*m*_*’*). PSII effective quantum yield (Φ*PSII*), non-photochemical quenching (*NPQ*) and relative electron transport rate (*rETR*) were calculated with Eqs 1, 2 and 3, respectively. According to [16], the Φ*PSII* can also be considered as the light utilization efficiency (LUE).

$$NPQ=(F_{m}/F_{m}\prime )-1$$ Eq 1

$$\Phi PSII=LUE=\left(F_{m}\prime -F\right)/F_{m}\prime$$ Eq 2

rETR=LUE×E Eq 3

### Spectroradiometry

At the end of each PAM measurement a volume of 0.5 mL diatom suspension was sampled, immediately diluted in 5 mL of artificial seawater and deposited on anisopore^TM^ polycarbonate membrane filter (Isopore^TM^1.2 μm, 25 mm RTTP filters, Merck Millipore, Darmstadt, Germany) by slow filtration limiting degradation of cells [27,28]. The filtration was done in the dark, during less than 30 sec. allowing the cell pigment content to remain similar as in the cuvette. This 5 mL volume allowed to homogenously cover membrane filters with a ~ 13 mg Chl *a*.m^-2^ layer, a representative biomass value encountered at the mudflat surface [12]. This value remains below saturation threshold occurring for values greater than 40 mg Chl *a*.m^-2^ [12]. All spectral measurements were performed immediately after filtration on wet membrane filters deposited over a black background. This precaution allowed avoiding multiple background reflectances which could trigger the xanthophyll cycle if the background is highly reflected as observed in pre-experimentation using *Navicula phyllepta* (data not shown). Five measurements per membrane filter were performed using an ASD FieldSpec3spectrometer (300–2500 nm, spectral resolution: 1 nm in the VIS-IR domain, property of the Laboratory of Planetology and Geodynamic (LPG-UMR 6112 of the University of Nantes) to determine radiance (mW.cm^-2^.nm^-2^.sr^-1^). The light source was provided by an internal halogen lamp (300–2500 nm) and the distance between the membrane filter and the ASD optical fiber was kept constant by using the ASD High Intensity Contact Probe. The distance of the lamp was 1.38 cm, the distance and viewing angle of the ASD fiber were respectively 0.76 cm and 55°. The field of view of the fiber with the contact probe was an oval (10.9 X 13.4 mm) smaller than the diameter of filter (25 mm). This procedure assured that only the cells were being measured and minimized any errors associated with stray light. Reflectance (*ρ*, dimensionless) was calculated as the ratio between the radiance of the cells on membrane filters and the incident radiance measured on a perfect diffuser (Spectralon® 99%). Reflectance was standardized (*ρ*_*std*_) to reflectance value at 925 nm, known to be invariable with diatom biomass to facilitate comparisons between spectra [28,29].

Second derivative of reflectance spectra (*δδ*) were calculated following Jesus et al. [17] and second derivative peaks were assigned to pigment absorption properties according to [29]. To facilitate the comparison between spectra, the second derivative values were standardized (δδ_std_) to the maximum value between 620 and 640 (*δδ*_*Chl c*_), corresponding to Chl *c* maximum red absorption.

Several radiometric indices were calculated with the objective of estimating LUE and rETR by radiometry. These indices were calculated using *in vivo* second derivative spectra absorption features and absorption properties previously published [17,30–32]. For a first set of indices, the biomass effect was removed by using Chl *c* absorption band (*δδ*_*Chl c*_) as suggested by [17]. However, because Chl *c* amount could vary between species for a similar light environment and could lead to ratio variations due to species and not to light condition, a second set of indices were constructed using only pigments absorption band involved in the xanthophyll cycle: the diadinoxanthin (DD) and its de-epoxidized form, the diatoxanthin (DT).

Each index was tested to predict LUE. Predicted values of LUE were compared to measured ones by PAM-fluorometry (Eq 2) for a new data set, using linear regression. Selected indices (see Data processing and model validation) were used to calculate rETR (Eq 3) and absolute ETR (Eq 4) needing the optical cross-section values, a* (m^2^.mg Chl a^-1^). The latter was retrieved from reflectance spectra using the radiative transfer model MPBOM (MicroPhytoBenthos Optical Model [27]) as proposed by [29]. Because background reflectance is needed to estimate a*, reflectance of a wet membrane filter with 5 mL of artificial seawater was measured at the beginning of each series of measurement.

$$ETR=rETR\times a^{*}$$ Eq 4

With a* corresponding to the average optical cross-section in the red domain between 670 and 685 nm Chl *a* absorption (= Qy band) [29,33,34].

### Pigment analyses by High Performance Liquid Chromatography (HPLC)

After each PAM and radiometry measurement, 1mL was filtered (Isopore^TM^1.2 μm, 25 mm RTTP filters, Merck Millipore, Darmstadt, Germany) and immediately frozen in liquid nitrogen for pigment analysis. Pigments were extracted in a cold mixture (4°C) of 90%methanol/0.2 M ammonium acetate (90/10 vol/vol) and 10% ethyl acetate. Injection, HPLC device (Hitachi Lachrom Elite, Tokyo, Japan), pigment identification and quantification [35] is detailed in [19]. All pigments were normalized to Chl *a* content (g.g Chl *a*^-1^). Xanthophyll de-epoxidation state was calculated following Eq 5.

$$DES=\left(DT-{DT}_{0}\right)/\left(DT+(DT-{DT}_{0})\right)\times 100$$ Eq 5

where DD is the epoxidized diadinoxanthin, DT is the de-epoxidized diatoxanthin after 5 min light exposure and DT_0_, the amount of DT before light exposure. DES calculated this way takes into account the de-epoxidation of DD into DT that specifically occurs during the 5 min light exposure of the NSLC.

### Data processing and model validation

All data are availed online: 10.6084/m9.figshare.5615746. The construction of the model to predict LUE and ETR by radiometric indices was achieved using measurements performed on *Navicula phyllepta* and *Biremis lucens* cultures, respectively an epipelic and an epipsammic growth form. Model significance was tested by linear regression (R software) and only radiometric indices explaining more than 40% (R^2^>0.4) of LUE variability measured by PAM fluorometry were kept. Validation of these selected models was carried out by comparing LUE measured by PAM-fluorometry with LUE predicted by radiometric indices for an independent data set from *Entomoneis paludosa*, *Planothidium delicatulum* and *Plagiogrammopsis vanheurckii* cultures, respectively an epipelic, an epipsammic and a tychoplanktonic growth form. Models with the smaller root mean square errors (RSME ≤ 0.02) were thus used for further calculation of ETR (Eq 4).

To demonstrate the viability of using the selected reflectance index to predict ETR on hyperspectral images, each diatom spectrum was degraded at several spectral resolutions from 3 to 15 nm in the NIR-VIS domain (400–1000 nm). The objective was to simulate reflectance spectra that would be obtained from existing or incoming hyperspectral sensors to calculate second derivatives [17] and index values, and to retrieve a* using the MPBOM. No hyperspectral images were used, but reflectance index values and a* were calculated from simulated spectra of various sensors to predict ETR. Spectral resolution simulation was carried out using ENVI software by convolving the reflectance data with Gaussian-like spectral response profiles. Actual spectral response profiles of varied sensors were used to generate a series of generic bandwidths from 3 to 15 nm FWHM (Full Width at Half Maximum): HySpex, AVIRISng, EnMAP, AVIRIS, Hyperion and HyMap. Spectral resolution where estimated ETR significantly fitted to ETR measured by PAM-fluorometry (R^2^ > 0.80; p<0.001) were considered strong enough to be used for monitoring MPB photosynthesis from Space.

## Results

### Xanthophyll cycle pigments

**Fig 2:**
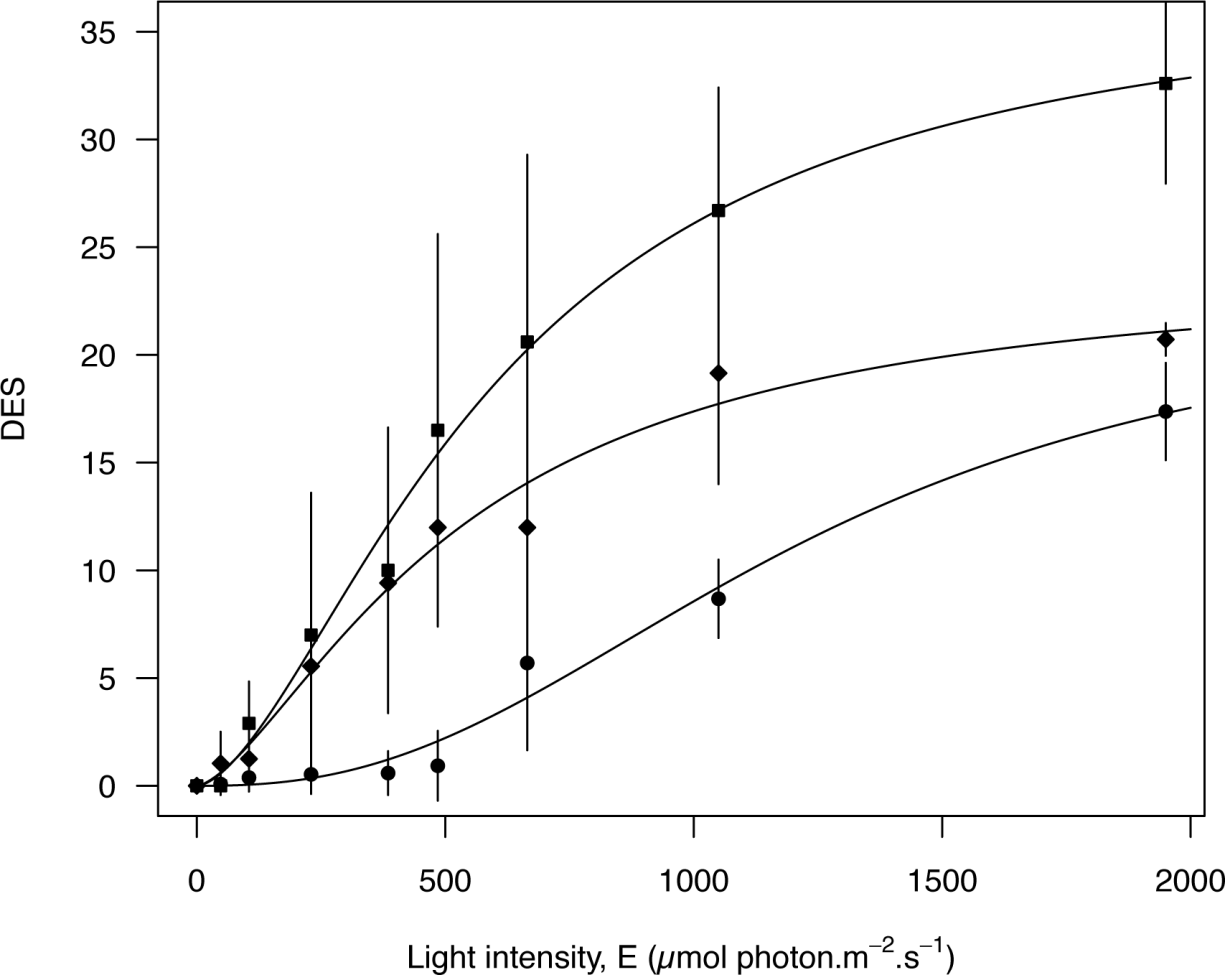
DES *versus* light intensity after 5 min exposure for the three growth forms. ●Epipelic (*Navicula phyllepta* and *Entomoneis paludosa*); ■Epipsammic (*Biremis lucens* and *Planothidium delicatulum*); ◆Tychoplanktonic (*Plagiogrammopsis vanheurckii*). Averaged DES were fitted using the model proposed by [37], vertical bars represent standard deviation.

All diatom main pigments (Table 1), Chl *c*, fucoxanthin (Fuco), *β*-carotene and xanthophylls (DD+DT) showed stable pigment to Chl *a* ratios during light exposures, but varied with species (2 way ANOVA: p>0.2 for light exposure; p<0.001 for species). The only pigment ratio that changed with light intensities was the xanthophyll cycle DES ratio estimating the conversion of the DD into DT with increasing light intensity (Fig 2). As expected (see [19]), the epipelic growth form showed the lowest DES values (17.37% ± 2.26 SD) together with the tychoplanktonic form (20.72% ± 0.76 SD). Epipsammic growth form showed the highest DES value (32.6% ± 4.65 SD). The stability in the pool of xanthophyll pigments (DD+DT) indicated that no DT was synthesized ‘*de novo*’ during illumination (see [36]) and only arose from the de-epoxidation of the DD.

**Table 1:** Pigment ratios relative to chlorophyll *a* of each strain, expressed in g.g^-1^ Chl *a*. Values are the mean of ratios obtained at the end of each light exposure (from 0 to 1950 μmol photon.m^-2^.s^-1^) ± variation coefficient. Growth form is presented for each strain.

	Chl *c*	Fuco	*β* car	DD+DT
*Navicula phyllepta* (epileplic)	15.41 ± 0.03	58.93 ± 0.01	3.78 ± 0.04	7.58 ± 0.06
*Biremis lucens* (epipsammic non-motile)	15.26 ± 0.02	56.80 ± 0.02	1.14 ± 0.22	9.68 ± 0.08
*Entomoneis paludosa* (epipelic)	18.91 ± 0.01	73.52 ± 0.01	3.59 ± 0.06	6.74 ± 0.04
*Planothidium delicatulum* (epipsammic motile)	17.57 ± 0.09	59.59 ± 0.09	1.25 ± 0.14	9.37 ± 0.10
*Plagiogrammopsis vanheurckii* (tychoplanktonic)	27.18 ± 0.05	92.78 ± 0.07	1.27 ± 0.2	9.95 ± 0.21

### Light use efficiency estimation based on PAM fluorescence

**Fig 3:**
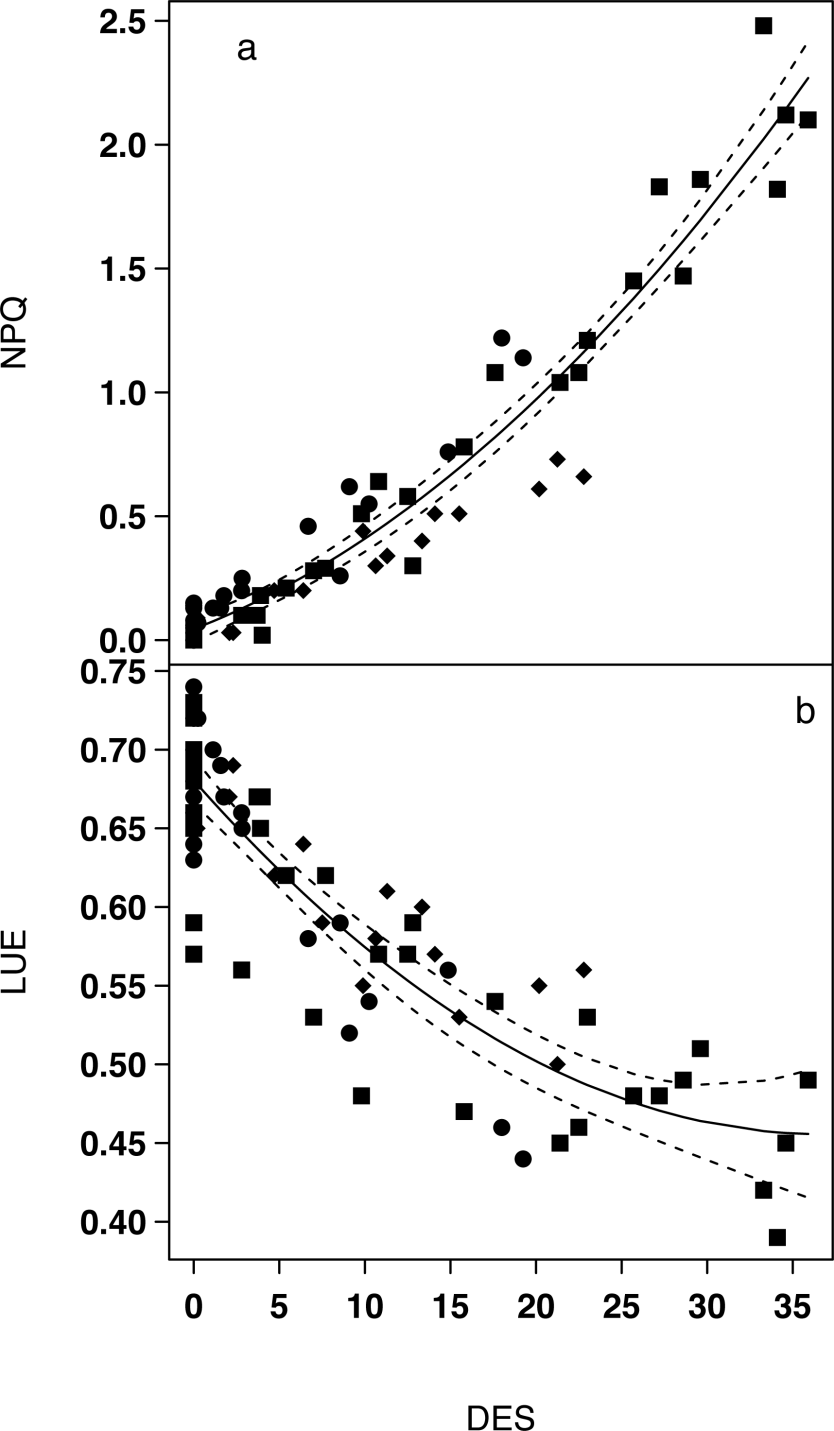
**Relationships between NPQ and DES (a) and LUE and DES (b) for the three growth forms.** ●Epipelic (*Navicula phyllepta* and *Entomoneis paludosa*); ■Epipsammic (*Biremis lucens* and *Planothidium delicatulum*); ◆Tychoplanktonic (*Plagiogrammopsis vanheurckii*). Equations of non-linear regressions are reported in the text (Eqs 8 and 9). Dashed lines represent 95% CI.

LUE and NPQ measured by PAM-fluorometry changed inversely with E: while NPQ increased, LUE decreased with increasing E. Consequently, LUE and NPQ showed inversed relationships with DES (Fig 3) and both could be predicted using DES (Eq 6 and Eq 7):

$$NPQ=0.001\times {DES}^{2}+0.026\times DES+0.046\;(R^{2}=0.94,\;p<0.001)$$ Eq 6

$$NPQ=0.0002\times {DES}^{2}-0.012\times DES+0.679\;(R^{2}=0.78,\;p<0.001)$$ Eq 7

The trend observed for the DES as function of light intensity (Fig 2) was kept for NPQ and LUE: epipsammic species were those with the highest NPQ and lowest LUE values, whereas epipelic and tychoplanktonic species showed inverted trends (Fig 3). Maximum NPQ values were reached by *B*. *lucens* (more than 2.0) for the highest light intensity corresponding to the highest DES (Fig 3A). LUE lowest values (≤ 0.5) were mainly observed in the two epipsammic species (*B*. *lucens* and *P*. *delicatulum*), even if *N*. *phyllepta* also showed low LUE at high light intensities (Fig 3B). Thus, growth forms responses showed a pattern along the regression curve: first epipelic and tychoplanktonic forms, then epipsammic ones for the highest DES level, never reached by the other growth forms (Fig 2).

### Selection of relevant indices from second derivative spectra

**Fig 4:**
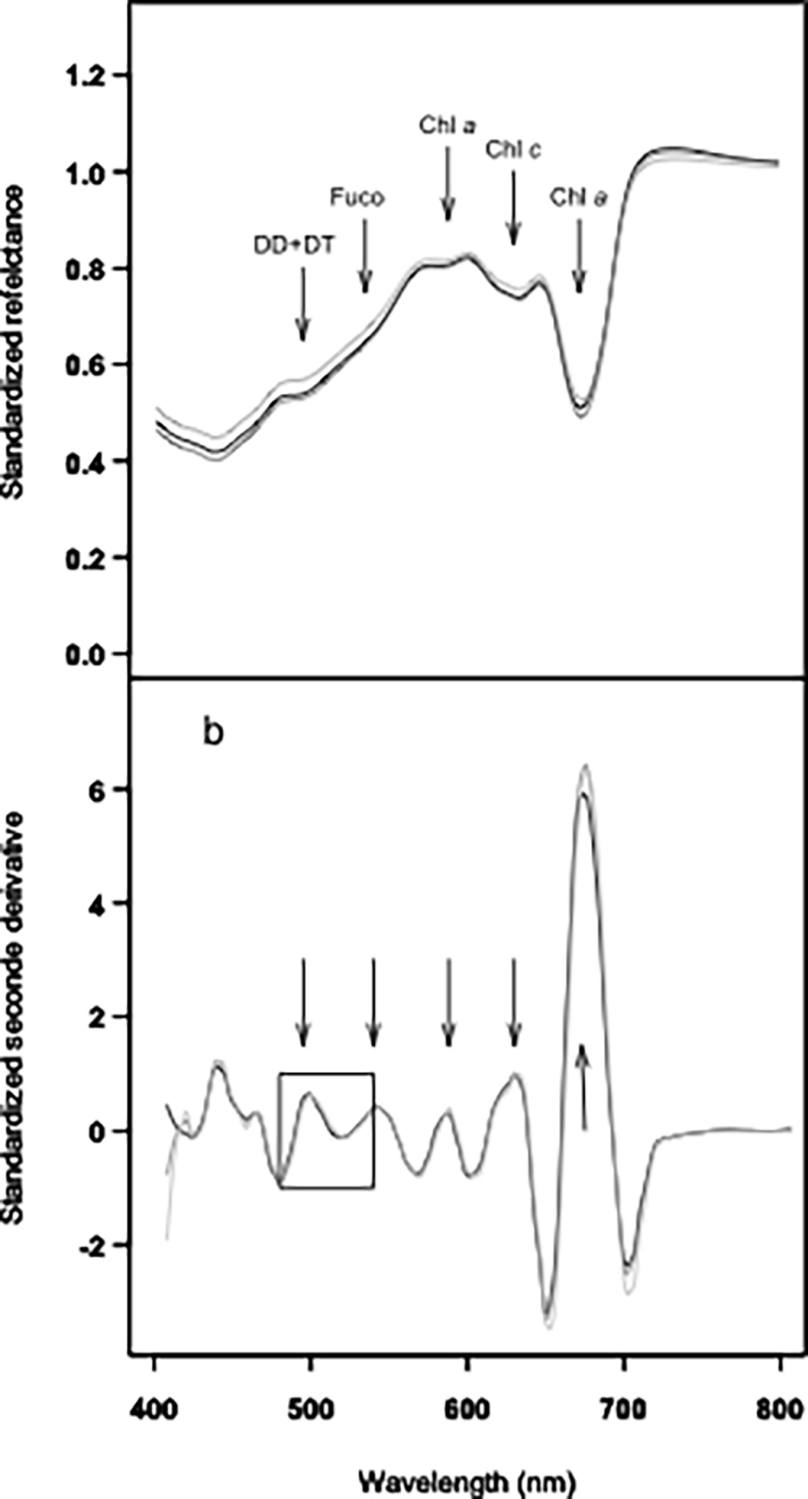
Typical radiometric spectra from *B*. *lucens* cultures exposed to three different light intensities. (5 min): 0 (black line), 665 (grey line) and 1950 (clear grey line) μmol photons.m^-2^.s^-1^. a/ Standardized reflectance; b/ Standardized second derivative. Arrows show absorption bands at 496, 540, 588, 632 and 673 nm respectively due to DD+DT, Fuco, Chl *a*, Chl *c* and again Chl *a* (see text and [29]). The box delimits the absorption domain due to DD and DT xanthophylls (Fig 5).

**Fig 5:**
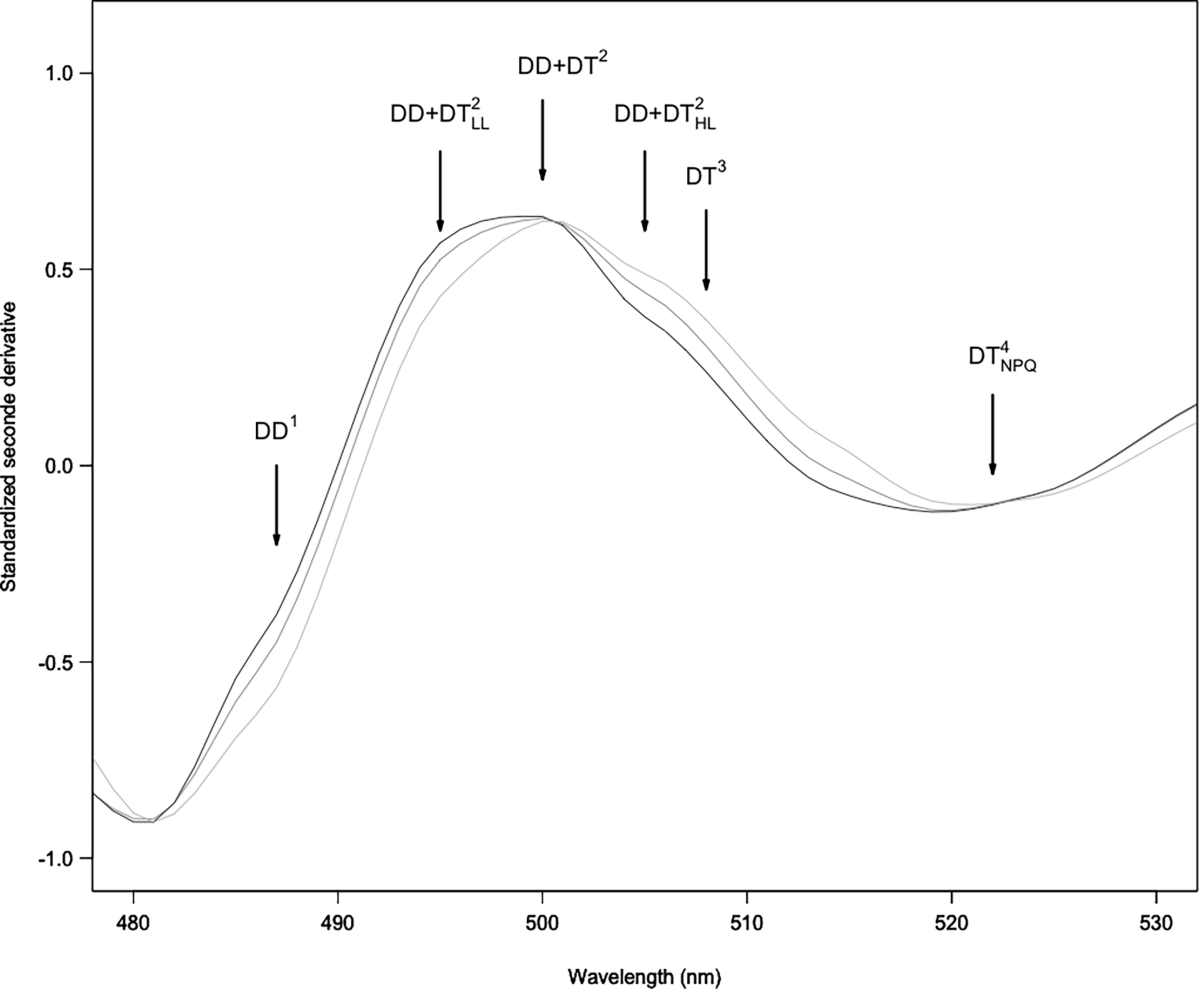
Zoom from Fig B for standardized second derivative over the absorption domain due to xanthophyll pigments involved in the XC (DD and DT) between 480 and 530 nm. Shoulders are assigned to absorption features of DD and DT from literature (DD^1^: [30]; DT^3^: [17]; and DT^4^_NPQ_: [31,38] and from this study (DD+DT^2^_LL_, DD+DT^2^ and DD+DT^2^_HL_). For details see text.

Spectral reflectance showed typical diatoms signatures [17,28] with specific pigment absorption bands respectively due to DD+DT (at 496 nm), Fuco (at 540 nm), Chl *c* (at 632 nm) and Chl *a* (at 588 and 673 nm) (Fig 4A). These absorption bands were confirmed using standardized (to Chl *c*) second derivatives spectra (*δδ*_*std*_, Fig 4B). However, DD+DT absorption band (around 496 nm) showed further absorption features (i.e. shoulders) in 2^nd^ derivative spectra, changing with light intensity as illustrated for *B*. *lucens* (Fig 5). Shoulders at 496, 500 and 505 nm were respectively assigned to DD+DT with a maximum during low light exposure (496 nm, DD+DT_LL_), no change with light at 500 nm, and DD+DT_HL_ increasing with light exposure (Fig 5). Shoulders at 487, 508 and 522nm were assigned to xanthophyll absorptions features from literature: DD^1^ according to [30], DT^3^ according to [17] and the ‘activated’ DT (DT^4^_NPQ_), i.e. the molecules of DT effectively involved in NPQ [31,38] (Fig 5). Note that under our NSLC light conditions (i.e. 5 min exposure), DT arose from the xanthophyll cycle only (i.e. DD de-epoxidation), DT was synthesized from DD de-epoxidation and no DT was synthesized ‘*de novo*’ [36].

These spectral features were used to establish spectral indices built up as band ratios. Some indices were standardized to the Chl *c* absorption at 632 nm. Linear relationships were calculated between each index and LUE values measured by PAM-fluorometry for two growth forms: epipelic (represented by *N*. *phyllepta*) and epipsammic (represented by *B*. *lucens*). Thirteen indices displayed significant relationships (R^2^> 0.4; p ≤ 0.001; Tables 2 and 3). Five fitted to both growth forms: *δδ*_508/632_; *δδ*_496/505_; *δδ*_496/508_; *δδ*_500/505_ and *δδ*_500/508_, seven fitted only to the epipsammic growth form: *δδ*_496/632_; *δδ*_520/632_; *δδ*_496/500_; *δδ*_496/520_; *δδ*_500/520_; *δδ*_505/520_ and *δδ*_508/520_; one index, *δδ*_505/632_, fitted only to the epipelic growth form. Within these 13 linear regressions, only 6 indices (Tables 2 and 3, in bold) allowed to predict LUE values of the three other species (*E*. *paludosa*, *P*. *delicatulum* and *P*. *vanheurckii*) with RSME values ≤ 0.02 (Eqs 8–13):

$$LUE=-3.130\times \delta \delta_{505/632}+1.702$$ Eq 8

$$LUE=1.597\times \delta \delta_{496/632}-0.347$$ Eq 9

$$LUE=-5.313\times \delta \delta_{520/632}+0.027$$ Eq 10

$$LUE=0.056\times \delta \delta_{496/508L}+0.247$$ Eq 11

$$LUE=0.064\times \delta \delta_{496/508S}+0.258$$ Eq 12

$$LUE=0.190\times \delta \delta_{500/520}+1.316$$ Eq 13

**Table 2:** Spectral indices calculated using second derivative value standardized to the Chl c red absorption band (*δδ*_632_) and explaining more than 40% of the variability (R^2^ > 0.4) of the LUE estimated by PAM-fluorometry using *Navicula phyllepta* (*N*. *phyl*) and *Biremis lucens* (*B*. *luce*) data set. The lowest values of RSME (= Root Mean Square Error), in bold, to predict LUE for *Entomoneis paludosa* (*E*. *palu*) *Planothidium delicatulum* (*P*. *deli*) and *Plagiogrammopsis vanheurckii* (*P*. *vanh*) are those selected (Eqs 8 to 13). ***: linear regression p ≤ 0.001; n.t.: not tested.

	R^2^		RSME		
	*N*. *phyl*	*B*. *luce*	*E*. *palu*	*P*. *deli*	*P*. *vanh*
***δδ***_**496/632**_	< 0.4	0.49***	n.t	**0.01**	0.28
***δδ***_**505/632**_	0.51**	< 0.4	**0.01**	n.t	0.03
*δδ*_508/632_	0.61***	0.73***	0.05	0.12	n.t
***δδ***_**520/632**_	< 0.4	0.61***	n.t	**0.01**	0.11

**Table 3:** Spectral indices calculated using second derivative from DD-DT xanthophyll pigments absorption band (see Fig 5) and explaining more than 40% of the variability (R^2^ > 0.4) of the LUE estimated by PAM-fluorometry using *Navicula phyllepta* (*N*. *phyl*) and *Biremis lucens* (*B*. *luce*) data set. The lowest values of RSME (= Root Mean Square Error), in bold, to predict LUE for *Entomoneis paludosa* (*E*. *palu*) *Planothidium delicatulum* (*P*. *deli*) and *Plagiogrammopsis vanheurckii* (*P*. *vanh*) are those selected (Eqs 8 to 13). ***: linear regression p ≤ 0.001; n.t.: not tested.

	R^2^		RSME		
	*N*. *phyl*	*B*. *luce*	*E*. *palu*	*P*. *deli*	*P*. *vanh*
*δδ*_496/500_	< 0.4	0.61***	n.t	0.05	n.t
*δδ*_496/505_	0.42**	0.74***	0.14	0.11	n.t
***δδ***_**496/508**_	0.47**	0.71***	0.13	**0.00**	**0.01**
*δδ*_496/520_	< 0.4	0.40***	n.t	0.3	n.t
*δδ*_500/505_	0.42**	0.76***	0.11	0.12	n.t
*δδ*_500/508_	0.44**	0.85***	0.09	0.26	n.t
***δδ***_**500/520**_	< 0.4	0.72***	n.t	**0.02**	0.15
*δδ*_505/520_	< 0.4	0.85***	n.t	0.04	n.t
*δδ*_508/520_	< 0.4	0.90***	n.t	0.06	n.t

Most of these six indices were species specific (Tables 2 and 3), except *δδ*_496/508_ which could be used for both *N*. *phyllepta* and *B*. *lucens* (Tables 2 and 3, Eqs 11 and 12). Analyses of covariance were performed with all the six regressions and confirmed that only those involving *δδ*_496/508*L*_ and *δδ*_496/508*S*_ were not significantly different (ANCOVA, p>0.05), and therefore not be affected by species. For this reason, we propose a single relationship based on the index *δδ*_496/508_ to predict LUE for all species and corresponding growth forms:

$$LUE=0.049\times \delta \delta_{496/508}+0.317$$ Eq 14

Since this index was not affected by species, it was selected for ETR prediction and hereafter called the MPB_LUE_ index.

### Electron transfer rate prediction using MPB_LUE_ index

**Fig 6:**
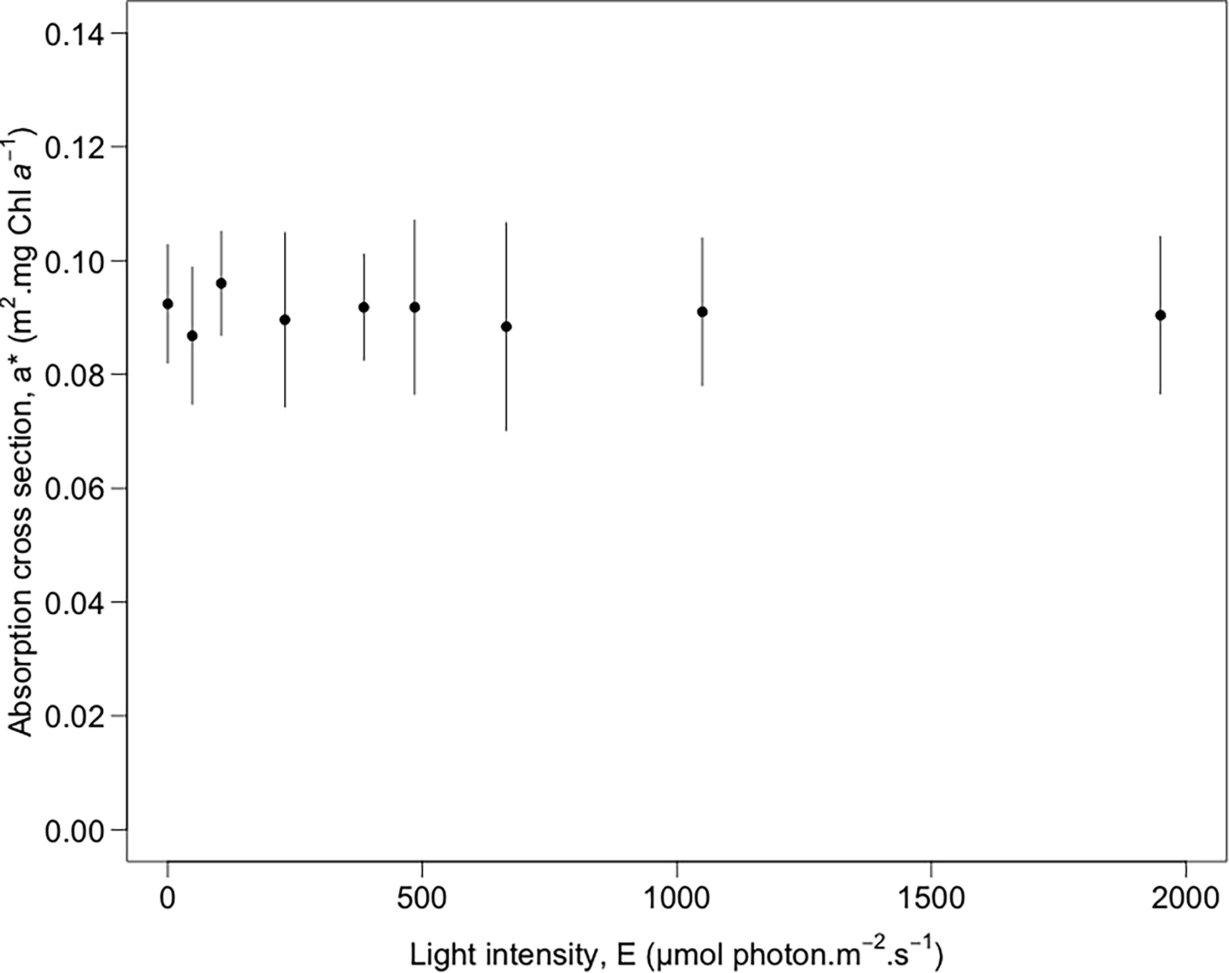
Optical absorption cross-section a*. Retrieved from the MPBOM transfer radiative model [27] and averaged over the Chl *a* absorption domain (670 to 685 nm) and for all species. Vertical bars represent standard deviation.

**Fig 7:**
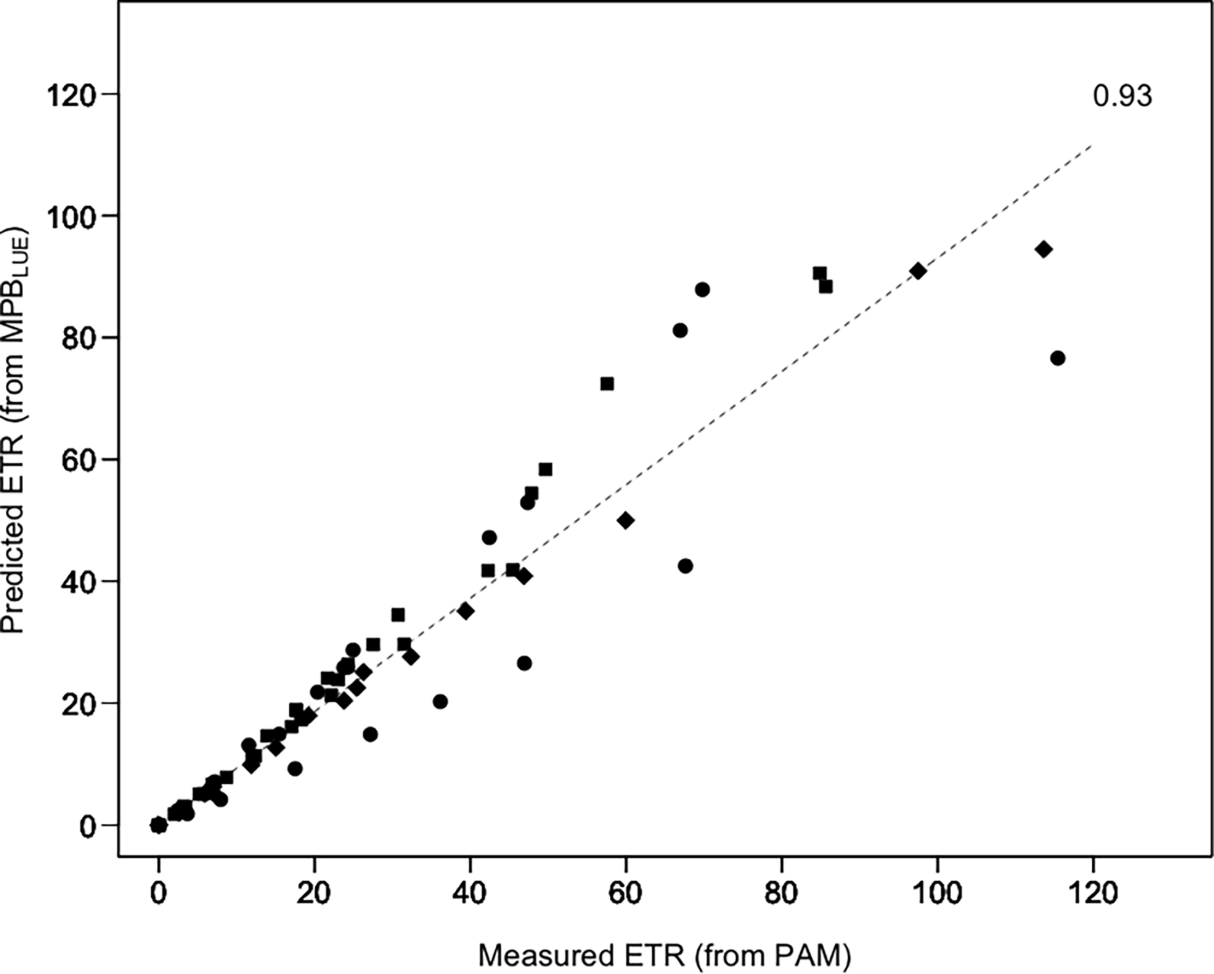
Measured ETR (from PAM fluorometry) *vs*. predicted ETR (from radiometric measurements using the MPB_LUE_ index). The dash line is the slope (= 0.93) of the linear regression (R^2^ = 0.92, p<0.001). All species and growth forms were included: ●Epipelic (*Navicula phyllepta* and *Entomoneis paludosa*); ■Epipsammic (*Biremis lucens* and *Planothidium delicatulum*); ◆Tychoplanktonic (*Plagiogrammopsis vanheurckii*).

ETR prediction following Eqs 3 and 4, needed the estimation of LUE by radiometry (from Eq 14), but also the optical absorption cross-section (a*) retrieved from the radiative transfer model MPBOM applied to each reflectance spectrum. We verified that the a* parameter remained stable at all light levels for all diatom species with a mean value of 0.09 m^2^.mg Chl*a*^-1^ ± 0.01 SD (ANOVA, p = 0.9, Fig 6). ETR predictions based on the MPB_LUE_ index showed a highly significant linear relationship with measured PAM-fluorometry ETR (R^2^ = 0.92, p<0.001; Fig 7) with a slope of 0.93 not significantly different from 1 (p>0.05).

### MPB_LUE_ application to hyperspectral sensors

**Fig 8:**
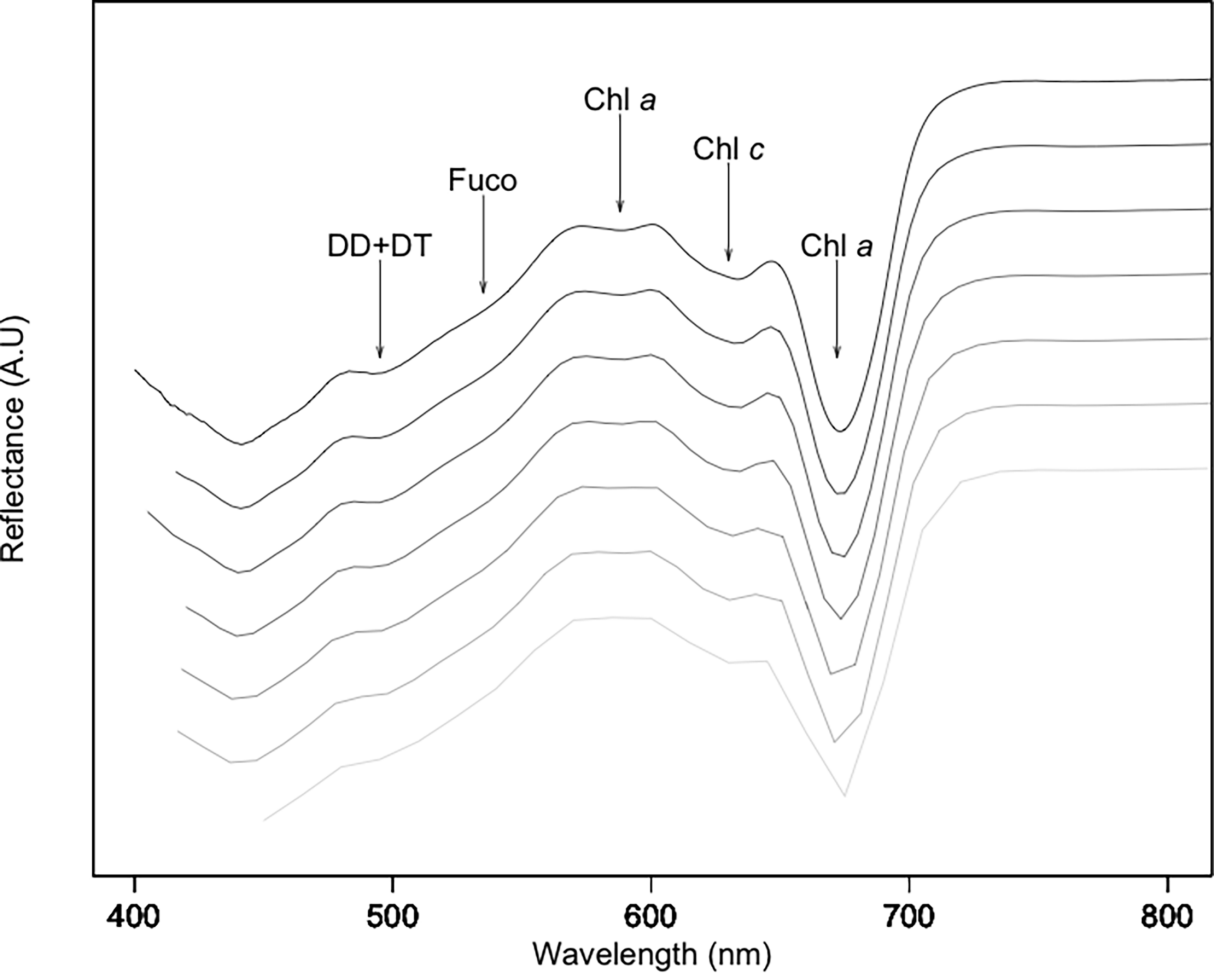
Spectra from *B*. *lucens* culture, after 5 min in the dark at different spectral resolutions. From the top (dark line) to the bottom (clear grey line): 1 nm (original data from ASD), 3.26 nm (HySpex simulation), 3.5 nm (CASI simulation), 6 nm (EnMap simulation), 10 nm (AVIRIS, Hyperion and HypXim simulation) and 15 nm (DAIS and HyMap simulation). Specific absorption features around 496, 540, 588, 632 and 673 nm respectively due to DD+DT, Fuco, Chl *a*, Chl *c* and again Chl *a* were still observable. Reflectance is presented in arbitrary unit (A.U) to avoid overlaying of spectra.

Reflectance spectra were degraded to 5 nm, 8.1 nm, 9.7 nm, 10.94 nm and 15.5 nm spectral resolution to simulate spectral responses from several sensors: airborne or satellite platforms, e.g. HySpex or Hyperion, and from upcoming sensors as EnMap (Table 4). Spectra global shape was only weakly affected by spectral degradation (Fig 8) and specific absorption features around 496, 540, 588, 632 and 673 nm were still observable, respectively due to DD+DT, Fuco, Chl *a*, Chl *c* and again Chl *a*. Lowest resolution (9.7 nm, 10.94 nm and 15.5 nm) smoothed spectra gave smaller a* values (0.08 ± 0.01) than those obtained with highest resolutions (0.09 ± 0.01) (Tukey’s pairwise p < 0.01) (Table 4). Second derivative wavelength values used to calculate MPB_LUE_ values for simulated sensors are reported in Table 4 for comparison with the original ones, i.e. 496 and 508 nm. For the three sensors with highest resolution and spectral sampling, two indices were tested because two spectral bands were close to 508 nm (Table 4). Predicted ETR from a* and MPB_LUE_ retrieved from simulated spectra were compared to ETR calculated from PAM fluorometry (slope, Table 4). Whatever the spectral resolution, linear regression slopes between ETR from PAM and from MPB_LUE_ were different from 1 (ANCOVA, p < 0.05) and decreased with spectral resolution. This illustrated the smoothing due to increasing FWHM. Regression coefficients were still high (R^2^ > 0.90) and significant (p < 0.001) except for indices based on wavelength higher than 508 nm: 495.2/509.6 (HySpex), 495/510 (AVIRISng) and 498/511 (EnMap). This illustrated the importance of absorption band location for indices calculation. However, index using the broader band sensor at 15.5 nm resolution (HyMap) remained useful in spite of the location of the band at 510 nm. This result could be explained by the integration of spectral response at 508 nm and above in this large spectral band whereas it was not the case for the smaller bands of high spectral resolution sensors.

**Table 4:** Spectral resolution (full width at half maximum, FWHM) and corresponding sensors used to simulate new spectra and retrieve new a* and MPB_LUE_ values for estimating ETR. Slope and R^2^ of linear regression between ETR estimated from fluorometry and radiometry are reported. All regressions are significant (p < 0.001), except ^(1)^; ^(2)^ Future sensors; ^(3)^ Spectral sampling is indicated when different of the FWHM.

Spectral resolution (Spectral sampling)^(3)^ *Spectral range*	Sensors (Company)	Platform *(Spatial resolution)*	a* ± std	MPB_LUE_ bands	Slope (R^2^)
3.5 nm (3.6 nm) *416–992 nm*	HySpex 1600 (Norsk Elekto Optikk)	Airborne *(≤ 1 m)*	0.09 ± 0.01	495.2/509.6 495.2/506	3.02 (0.19) 0.82 (0.95)
5 nm *380–2510 nm*	AVIRISng	Airborne *(1–4 m)*	0.09 ± 0.01	495/510 495/505	0.99^(1)^ (0.14) 0.75 (0.92)
8.1 nm (6.5 nm) *420–1000 nm*	EnMAP^(2)^ (DLR)	Satelite *(30 m)*	0.09 ± 0.01	498/511 498/504.5	1.16 (0.23) 0.74 (0.98)
9.7 nm *for 360–670 nm* 9.5 nm *for 660–1280 nm*	AVIRIS (NASA)	Airborne *(1–4 m)*	0.08 ± 0.01	495/505	0.63 (0.94)
10.94 nm (9.2 nm) *355*.*59–2577*.*08 nm*	Hyperion (NASA)	Satelite *(30 m)*	0.07 ± 0.01	498/508	0.66 (0.97)
15.5 nm (15 nm) *450–1350 nm*	HyMap (HyVista)	Airborne *(5 m)*	0.08 ± 0.01	495/510	0.61 (0.97)

## Discussion

### Selection of a radiometric index for ETR prediction: The MPB_LUE_ index

The objective of this study was to find a radiometric index to predict ETR from reflectance spectra as a proxy for MPB primary production. The choice of PAM-fluorometry to estimate ETR to calibrate a radiometric index was based on the fact that data time acquisition is in the same time range for both techniques. Using NSLCs and other light curves [18], photosynthetic parameters and reflectance data are obtained in the same time range, i.e. less than 1 second for each measurement type [39]. This time range duration reflects similar processes: fluorescence emission time due to actinic light effect and the reflectance of this light. Alternative techniques require longer measuring times, e.g. CO_2_ fluxes using benthic chambers can take more than 20 min [40]; ^14^C based techniques take even longer [41], integrating processes that take much longer than pigment de-epoxydation or electron transfer. Furthermore, radiometric data are closely linked to pigment composition as any change in pigment content is known to induce reflectance and second derivative spectral changes [29,42]. Changes in chlorophyll fluorescence (especially via NPQ) is also closely related to pigment composition, namely to the xanthophylls DD and DT [17,43–45]. For these two reasons, i.e. time scale and strong relationship with pigments, PAM-fluorometry is probably the better technique to couple with spectroradiometry. Additionally, the choice of PAM-fluorometry to calibrate reflectance data with the purpose of estimating MPB primary production is also supported by the results from [46]. They observed correlation between MPB community production (measured with benthic chambers) and rETR (measured by PAM-fluorometry) at different sites and different seasons [46], even if the relationship could be site-dependent [47].

In this study, we focus on the light effect on ETR estimation whereas temperature also can influence the ETR and thus its relationship with MBP_LUE_. However, measurements were done at 20°C, near to the optimal temperature for MPB primary production [41], and it is known that MPB is adapted to a wide range of temperature without significant change in primary productivity [48].

From all the indices tested in this study only one index was selected, i.e. the MPB_LUE_ index (*δδ*_496/508_) because it was independent of species and growth forms. This is an unexpected result because diatom growth forms strongly affect eco-physiological response to light exposure as demonstrated previously [19–22]. It was confirmed by the present work with expected differences between epipsammon and epipelon DD de-epoxidation responses (i.e. highest and lowest, respectively, see [19]). Although DES differences were significant between growth forms, it did not affect ETR prediction using the MPB_LUE_ index. This result could be explained by the direct link between this index and the DES level: a high DES level corresponds to a low MPB_LUE_, whatever the growth forms. This is confirmed by the other indices based on xanthophyll absorption bands: these indices are still significant for all growth forms, but less than the MPB_LUE_. On the other hand, indices using Chl *c* absorption band are highly growth form dependent, due to Chl *c* content changing with species.

This is ideal for remote sensing applications since the MPB_LUE_ index could be applied to natural microphytobenthic assemblages independently of their growth form/specific composition as long as they are dominated by diatoms which is most often the case for temperate mudflats [2,49–51].

All indices investigated here were mainly linked to DD and/or DT absorption bands around 500 nm. Globally, absorption before 500 nm decreased with light whereas over 500 nm it increased. This pattern is due to the de-epoxidation of DD into DT as previously reported by [17]. It explains why several indices identified in this study are close to the one proposed by [17], i.e. *δδ*_508/632_. However, the relationship between second derivative wavelength and LUE was different: [17] described an exponential relationship, whereas we found linear one. This difference could be explained by: 1) light conditions (acclimation, intensity and duration) were different and responsible for range of LUE smaller in the current study; 2) Chl *c* content in species might have been different, leading to index variation not related to DES change but rather to a change in Chl *c* content due to species-dependent feature. This latter observation reinforces the use of the MPB_LUE_ index to predict ETR, because it is both species- and Chl *c*- independent.

The radiometric index based on the 522 nm absorption band was expected to be more robust because it has been shown to be a fingerprint for DT molecules effective in NPQ [31,38]. However, among the species used here, only *B*. *lucens* showed a strong relationship between PAM and radiometric indices using 522 nm band. The weakness of 522 nm-based indices in *N*. *phyllepta* data was likely due to the low DES and DT content, and the subsequent low NPQ [19]. Hence for some species, DT content might be too low to be detected by radiometry, a less sensitive method than the spectrophotometric approaches used before [31,38].

### Remote sensing applications

Remote sensing has been used to map MPB biomass, using multi- and hyperspectral imagers [9,11–14,52]. It was also used to estimate optical absorption cross section (a*) retrieved from optical properties of the MPB biofilm [27,29]. More recently, [15] suggested the use of remote sensing to estimate primary production from Space, but using passive chlorophyll measurements (solar-induced Chl fluorescence). However, issues related to spatial, spectral, and temporal dynamics of passive vegetation fluorescence are still unresolved and hinder this method [53]. The current study can be seen as an alternative for mapping MPB primary production using VIS-NIR remote sensing.

Current results suggest that MPB_LUE_ can be applied to existing hyperspectral sensors and any future sensors with higher than 10 nm resolutions. Nevertheless, some possible limitations and recommendations are discussed bellow: mixed assemblage effect, spectral resolution and spatial heterogeneity.

#### Mixed assemblage effect

An expected difficulty, but overcome in this study, is the species composition of natural MPB assemblages: sandy sediments are considered to be colonized by epipsammic assemblages, whereas muddy sediment by epipelic ones [49,50,54]. However, each assemblage can host other growth forms: e.g. epipelic growth forms in epipsammic assemblage or tychoplanktonic growth forms in epipelic assemblages [55]. This could lead to a mix of photophysiological response of each growth form [19] difficult to assess at the assemblage and ecosystem level. One possible difficulty to apply our methodology to field situation is the self-shading by cells within the biofilm and/or the migration of epipelic species vertical within a light gradient. The present work shows that this limitation can be partially overcome by using the MPB_LUE_ index as it is only affected by the xanthophyll pigments (DD and DT) and not by diversity, i.e. the MPB_LUE_ index appears to have a wide-ranging applicability regardless of the growth form type, as the NDVI for vegetation biomass. However, the next step is to test this index *in situ* taking into account not only the diversity, but also cell behavior within a biofilm.

#### Spectral resolution requirements

MPB_LUE_ was shown to work at several spectral resolutions of 15 nm and less suggesting that the signal is strong enough for detecting xanthophyll pigments absorbance variations between 496 and 508 nm. The spectral band location for MPB_LUE_ calculations is essential: they have to be as close as possible to 496 and 508 nm. Resolution higher than 9 nm resulted in significant linear regressions between ETR form PAM-fluorometry and from radiometry but with a clear ETR underestimation, due to the smoothing of signal, as for a* estimation. To compensate low spectral resolution, spectral bands must be located on the accurate wavelengths, with a bandwidth inferior or equal to 15 nm to avoid spectral band overlays.

#### Spatial heterogeneity

It is well known that MPB biofilms exhibit spatially patchy biomass distributions at both micro- and macro-scales [56] leading to non-linear reflectance mixing at the pixel scale [8,11]. This constraints remote sensing applications, because using linear model as indices (e.g. NDVI, MPBI, I_diatom_ [12]) could lead to misestimating biomass, LUE, and ETR when distribution of biofilm is too patchy. The best way to limit this misestimation is to work at very high spatial resolution (1 m^2^ or less) and at a very high spectral resolution, i.e. at least 10 nm with bands centered on the accurate wavelengths (see above). However, sensors with high spectral and spatial resolutions are currently only airborne, (e.g. HySpex, CASI or AVIRIS) and currently no satellite solution exists. Nevertheless some hyperspectral projects are planned for a near future that will approximate the needed requirements, such as the CNES (French Spatial Agency) project HypXim with a spatial resolution of less than 10 m and spectral resolution of 10 nm.

## Concluding remarks

Hyperspectral remote sensing is a highly promising technology to estimate MPB electron transport rates and subsequently estimate primary production at the ecosystem level. Here we showed how a new robust index, the MPB_LUE_ based on reflectance data in the DD and DT absorption domain, could be used to estimate ETR from hyperspectral imagery. We demonstrated that the species diversity analyzed in this study did not affect MPB_LUE_ index, and that it can most probably be applied to epipelic, epipsammic and tychoplankton species in mixture, i.e. in mixed sediments. We concluded that spectral resolution at 10 nm with bands centered around 496 and 508 nm and spatial resolution of m^2^ (1 m^2^ or less) is the minimal requirement needed to reach our goal: map MPB primary production. Meanwhile, further work is needed to validate the MPB_LUE_ index in field conditions (i.e. on natural MPB assemblages) including vertical migration of epipelic species as a response to incident light, but also to day/night and tidal cycles. The final step will be to thoroughly determine the relationship between reflectance, ETR and carbon fixation in order to build maps of carbon fluxes (mg CO_2._h^-1^.m^-2^) at the scale of entire mudflats.
